# Habitat integrity and canopy cover shape small-scale variation in coloration among odonate assemblages in Neotropical streams

**DOI:** 10.1007/s00442-026-06003-3

**Published:** 2026-10-25

**Authors:** Francisco Maciel Barbosa-Santos, Felipe Datto-Liberato, Rhainer Guillermo-Ferreira, Luciano Fogaça de Assis Montag, Thaisa Sala Michelan, Leandro Juen

**Affiliations:** 1 Laboratório de Ecologia e Conservação (LABECO), Instituto de Ciências Biológicas (ICB), Universidade Federal do Pará (UFPA), Rua Augusto Corrêa, nº 1, Bairro Guamá, Belém, Pará, CEP 66.075-110 Brazil; 2 Programa de Pós-graduação em Ecologia (PPGECO), Instituto de Ciências Biológicas, Universidade Federal do Pará, Belém, Pará Brazil; 3 Lestes Lab, Entomology and Experimental Biology Center, Federal University of Triangulo Mineiro, Uberaba, Brazil

**Keywords:** Amazonia, Anisoptera, Environmental gradient, Thermoregulation, Zygoptera

## Abstract

Small aquatic ecosystems, such as Amazonian igarapés, harbor high biodiversity composed of organisms essential for ecosystem functioning. Dragonflies and damselflies (Odonata) are abundant and sensitive to habitat alterations, making them useful indicators of environmental conditions. Their conspicuous diversity and coloration patterns support behavioral strategies and thermoregulation, communication, and protection against solar radiation and water loss. In this study, we evaluated whether environmental gradients influence Odonata species distribution in Eastern Amazonian streams through selection on coloration traits. Specifically, we tested (i) whether local environmental conditions are associated with assemblage-level variation in non-circular coloration traits (Saturation, Value, melanin coverage, and wax-derived pruinescence coverage); and (ii) whether species composition varies along gradients of habitat integrity and canopy cover. We sampled 25 streams and analyzed 213 individuals from 32 species/morphospecies. Using standardized photographs, we quantified body coloration in the HSV colour space (Hue, Saturation, and Value) and estimated melanin and pruinescence coverage. We analyzed community-weighted means of Saturation, Value, melanin coverage, and pruinescence coverage; we used Hue descriptively. Saturation and Value declined with habitat integrity; Value increased with canopy cover, whereas pruinescence coverage decreased. Melanin coverage showed no clear environmental association. More shaded streams unexpectedly supported assemblages with higher Value, and species composition varied with habitat integrity. These findings suggest that distinct coloration components, together with other physiological traits, may help shape fine-scale Odonata community composition along microclimatic gradients. Coloration may complement occurrence and abundance data in environmental assessment, pending experimental validation of the mechanisms involved.

**Supplementary Information:**

The online version contains supplementary material available at https://doi.org/10.1007/s00442-026-06003-3.

## Introduction

Land-use and land-cover changes, including the conversion of forests into monoculture plantations and pastures, as well as hydropower development, mining, and urbanization, profoundly alter environmental conditions (Rivera-Pérez et al. 2025; Cajaiba et al. 2018). These activities affect both terrestrial and aquatic ecosystems (Ball et al. 2010). In streams, the loss of riparian vegetation alters energy inputs, destabilizes the banks, increases erosion, and reduces habitat heterogeneity (Cruz et al. 2025). It also increases the amount of solar radiation reaching the channel, thereby raising water temperature (Silva et al. 2024; Faria et al. 2021).

Amazonian igarapés are small, shallow forest streams that form dense drainage networks and are strongly influenced by the surrounding riparian vegetation. These ecosystems support high taxonomic and functional diversity and contribute to essential ecological processes, including organic-matter decomposition, nutrient cycling, and energy transfer through aquatic food webs (Oliveira-Junior et al. 2021; Cruz et al. 2025).

Aquatic insects, due to their high specificity to environmental conditions, are excellent tools for bioindication (Juen et al. 2025). Among them, dragonflies and damselflies (Odonata) stand out because of their sensitivity to habitat alteration and their value as indicators of environmental conditions (Gerlach et al. 2013; Miguel et al. 2017; Valente-Neto et al. 2016; Mendoza-Penagos et al. 2021; Datto-Liberato et al. 2024). They live in freshwater habitats, such as rivers, lakes, ponds, and marshes (Mendes et al. 2018). Odonata spend much of their life cycle in water during the larval stage. As adults, they remain associated with aquatic habitats, where they forage, reproduce, and patrol territories important for reproduction (Corbet 1999).

Within a few days of their emergence from the water, adult Odonata exhibit a wide variety of body colors (Suárez-Tovar et al. 2022). Coloration can directly influence thermal performance, as darker coloration may increase the absorption of solar radiation during basking, facilitating body warming and the onset of daily activity (Moore et al. 2019). Environmental gradients such as canopy cover, habitat integrity, air temperature, and stream width may influence Odonata assemblages by altering solar exposure and local microclimatic conditions. These gradients can favor species with different coloration traits, including variation in hue, saturation, brightness values, melanin, and wax coverage, according to their thermoregulatory and physiological requirements (Barbosa dos Santos et al. 2024; Brito et al. 2024). Odonata show diverse colour patterns that may contribute to thermoregulation, communication, predator deterrence, and other ecological functions (Futahashi 2016; Salami et al. 2019).

Odonates regulate body temperature through behavioral and morphological mechanisms, including posture adjustment, habitat selection, body size, and variation in body and wing pigmentation (May 1976; Moore et al. 2019; Laakso et al. 2021). Darker pigmentation may increase solar radiation absorption and accelerate warming under cooler conditions, although its effects depend on body region, body size, species identity, ambient temperature, and radiation levels (Moore et al. 2019; Laakso et al. 2021). In addition to pigmentation, structural coloration and wax-derived optical properties may influence how odonate colour patterns function in communication and visual signalling, as shown for wing coloration in Zenithoptera lanei (Guillermo-Ferreira et al. 2015). Light availability can also influence habitat selection, with some forest-dwelling damselflies selecting shaded environments and avoiding highly illuminated habitats (Henry et al. 2018). Together, these findings indicate that coloration-related traits may vary along environmental gradients, but these patterns may also be a by-product of differences in solar radiation, microclimatic conditions, and habitat structure rather than direct evidence of species sorting by coloration. Therefore, the ecological effects of coloration are context-dependent and should be interpreted cautiously.

The coloration can be described by attributes such as hue (H), saturation (S), and value (V), which together define the HSV colour space and represent perceptual dimensions (Cantrell et al. 2010; Lopez et al. 2021). Hue (H) identifies the dominant chromatic category according to its position on a circular color scale, distinguishing colors such as red, yellow, green, blue, and violet. In our measurements, we expressed hue on a scale ranging from 0º-360º. Because Hue is circular, values close to 0° and 360° represent similar chromatic categories; therefore, Hue was interpreted only descriptively and was not included in the linear inferential analyses. Saturation (S) represents the intensity or purity of a color, whereas value (V) represents its relative lightness within the HSV colour space. Melanin coverage and wax derived pruinescence (henceforth pruinescence) coverage also comprise important functional traits: melanin is associated with dark pigmentation and may increase thermal absorption (Liu et al. 2016; Sun et al. 2024), whereas pruinescence may contribute to UV reflection, optical properties, surface hydrophobicity, and visual signalling in odonates (Gorb et al. 2000; Guillermo-Ferreira et al. 2015). Although Odonata coloration may also involve other pigmentary mechanisms, including pteridines and ommochromes, these pigments were not quantified in our study. Instead, we focused on HSV-based colour attributes, melanin coverage, and pruinescence coverage as standardized traits that could be measured from calibrated photographs. Despite the relevance of these coloration components, few studies have simultaneously investigated how environmental gradients are associated with multiple coloration traits and how these traits vary among Odonata assemblages, especially in tropical environments such as Amazonian streams.

Considering this scenario, we evaluated whether variation in the coloration of dragonfly species influences species distribution along the environmental gradient of streams in Eastern Amazonia. We tested two hypotheses: (i) local environmental conditions, particularly canopy cover, habitat integrity, and air temperature, are associated with variation in non-circular coloration traits, including Saturation, Value, melanin coverage, and pruinescence coverage; and (ii) species composition varies along the environmental gradient, especially in relation to forest cover and habitat integrity. We expected that more shaded and cooler streams would be associated with assemblages showing darker coloration and lower Value, because darker surfaces may favor heat absorption under reduced solar radiation. Conversely, more open and sunlit streams would be associated with assemblages showing higher Value and greater pruinescence coverage, reflecting potential complementary strategies related to thermoregulation and resistance to dehydration. Hue was retained only for descriptive interpretation because it is a circular component of colour and was therefore not included in the inferential trait–environment analyses.

## Materials and methods

### Study area

**Fig. 1:**
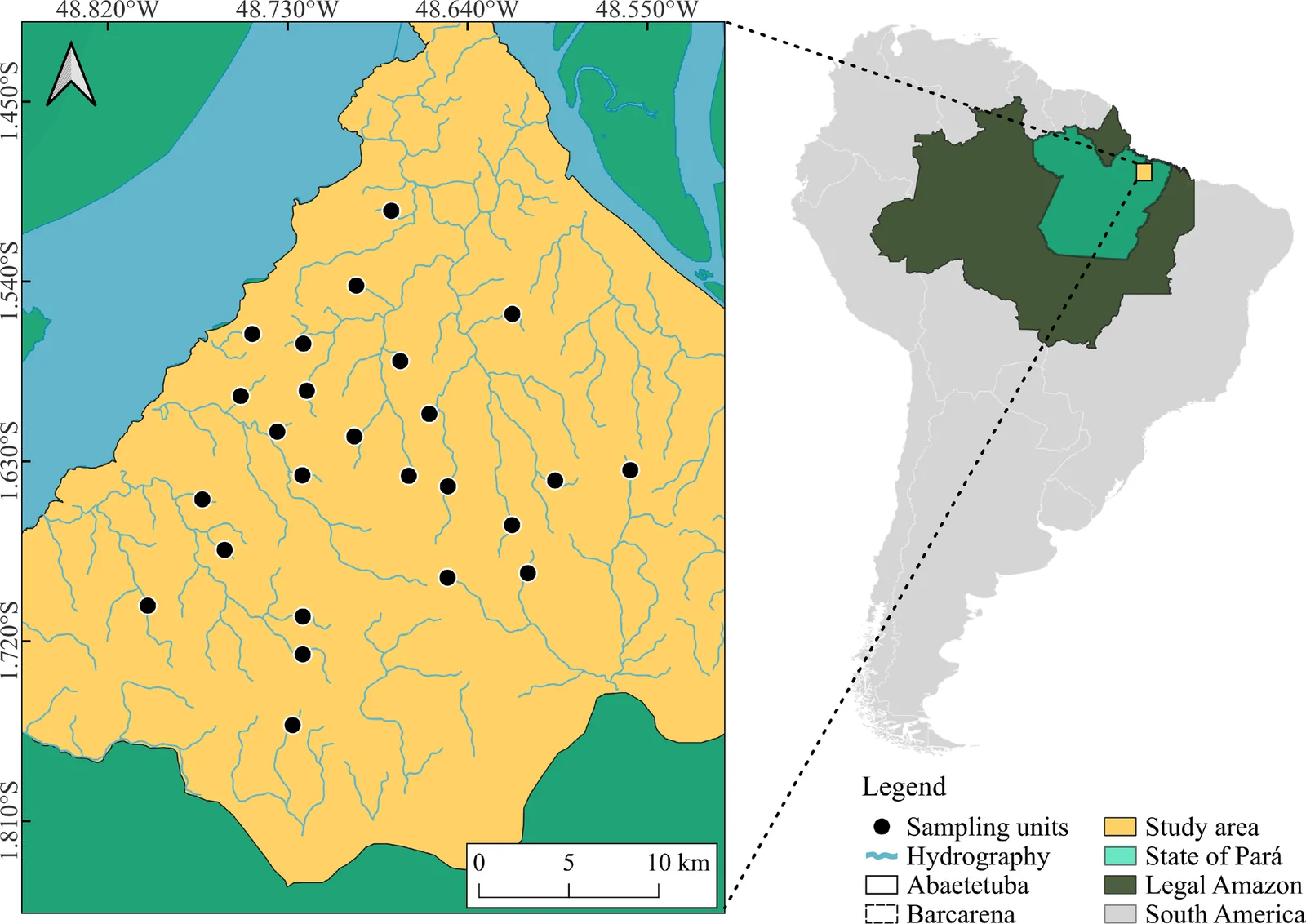
Map representing the 25 sampling sites distributed in the municipalities of Barcarena and Abaetetuba, in the state of Pará, Brazil

The study was conducted in the municipalities of Barcarena and Abaetetuba, in the state of Pará, northern Brazil (Fig. 1). The climate is equatorial, with mean annual precipitation ranging from 2.000 mm to 3.300 mm, a rainy season from January to June, a drier period from July to November, and mean annual temperatures ranging from 24 to 31 C° (Climatempo 2026).

The region presents an intense history of land use for short-cycle agriculture, livestock, industrialization, and urbanization activities (Paz et al. 2011; Furtado et al. 2020). These activities directly affect streams through waste deposition, alteration of stream banks, modification of vegetation cover, reduction in habitat integrity, and landscape fragmentation (Lemos and Pimentel 2021; Rivera-Pérez et al. 2025). The vegetation is mainly composed of subperennifolious equatorial forest, with variation in its appearance and structural characteristics (Cunha et al. 2022), and is classified by IBGE as dense terra firme forest or humid tropical forest. The sampled streams were selected to represent a regional gradient of land use and environmental integrity, ranging from environments without riparian forest due to anthropogenic disturbance to forested environments with a continuous canopy, which represent the best environmental condition possible in the region.

### Odonata sampling

Specimens of Odonata were collected from 25 streams between September and October 2024, during a period of low precipitation in the region. Each stream was represented by one continuous 150-m reach, and each stream was treated as an independent sampling unit (Cezário et al. 2021). Therefore, the study comprised 25 independent sampling units, with no pooling of multiple reaches within the same stream (Juen et al. 2025). In each segment, adult Odonata specimens were sampled using an entomological net with a 40 cm diameter, following the fixed-area sweeping methodology described by Juen and De Marco (2011) and Juen et al. (2014). The mean time spent at each sampling site was one hour, with collections always occurring between 10:00 and 14:00 h to ensure sampling of adult individuals during the period of greatest daily activity, when most Odonata species are active and thermoregulating (Oliveira-Junior et al. 2021). The abundance data used in the community analyses corresponded to the total number of individuals of each species or morphospecies collected within each 150-m reach during this standardized sampling period. Thus, the dataset represents a standardized temporal snapshot of adult assemblages during the low-precipitation period rather than complete seasonal variation in species occurrence.

### Coloration data collection

After sampling each stream, the live specimens were transported to a temporary field base established in Barcarena, where standardized photographs were taken on the same day, approximately 1–3 h after collection. This procedure minimized potential changes in body coloration while allowing all photographs to be obtained under standardized conditions. The digital images were captured with a Canon camera and saved in RAW format to allow for the use of image-processing software tools. This format reduces data loss due to pixel rounding and/or saturation, in addition to facilitating measurements of bright objects (Troscianko and Stevens 2015). The camera was mounted on a tripod, and all photographs were standardized with a white background. We used a *ColorChecker* containing 24 calibrated chromatic standards to standardize illumination and correct colors (see supplementary material Fig. 1). Images were calibrated using *Adobe Lightroom Classic*^®^ software (version 13.4) with the *ColorChecker* Camera Calibration plug-in (version 2.2.0). After calibration and standardization of the photographs of each individual, we used Paint software to extract HSV values. The HSV colour space is composed of three parameters: Hue (H), which represents the chromatic angle; Saturation (S), which represents colour intensity or purity; and Value (V), which represents relative lightness. Melanin coverage and pruinescence coverage were estimated separately as proportional surface-coverage traits and were not part of the HSV colour space. (Lopez et al. 2021; Guillermo-Ferreira et al. 2023).

**Fig. 2:**
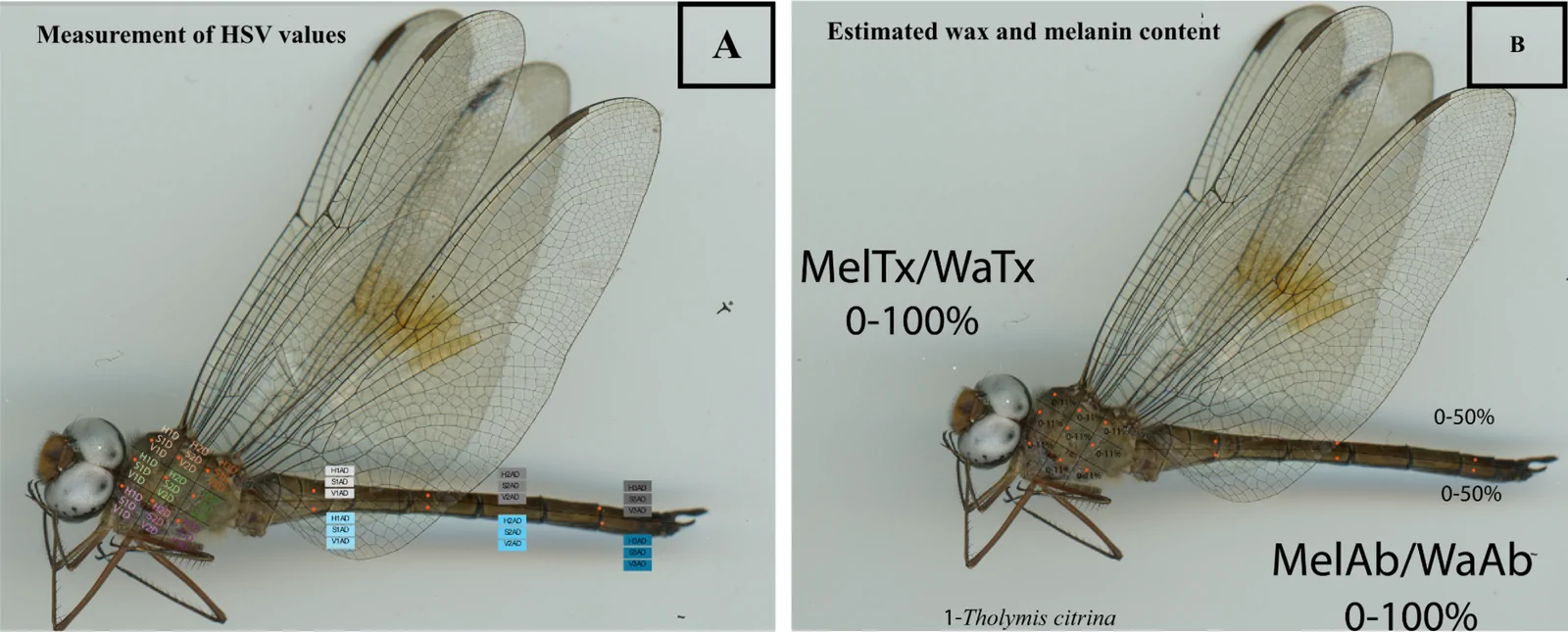
Standardized anatomical areas used to extract coloration data. (**A**) Extraction of HSV information from nine areas on the thorax and six areas on the abdomen. (**B**) Areas used to estimate melanin and pruinescence coverage; the percentage values indicate the estimated proportion of the standardized thoracic or abdominal measurement areas covered by melanin or pruinescence. Abbreviations: MelTx = thoracic melanin; MelAb = abdominal melanin; WaxTx = thoracic pruinescence; WaxAb = abdominal pruinescence; H = Hue; S = Saturation; V = Value

Color measurements were obtained from nine standardized points on the visible lateral surface of the thorax and six points on the abdomen (Fig. 2, panels A and B). These points were selected to cover the largest possible proportion of each body region while maintaining anatomical correspondence among individuals and avoiding overlap among measurement areas. The thoracic points were distributed across the anterior, central, and posterior portions of the synthorax, whereas the abdominal points were distributed along the proximal, medial, and distal segments (Lopez et al. 2021; Guillermo-Ferreira et al. 2023). The exact position of each point is shown in Fig. 2. The same set of anatomically corresponding points was used for every specimen. HSV values were not extracted from single pixels. Instead, they were measured from standardized anatomical areas centred on each point, allowing comparable estimates of coloration across individuals and species. Although this standardized area-based protocol allowed comparisons among individuals and species, it does not quantify all colour variation across the entire body surface. Future studies could improve this approach by using pixel-level segmentation or accumulation-curve analyses to determine the number of areas required to adequately represent whole-body coloration. Because Hue is a circular variable, in which values close to 0° and 360° represent similar colours, Hue was retained only for descriptive and visual interpretation.

Each sampled individual was photographed once under standardized conditions. When more than one individual was available for a species or morphospecies, trait values were first obtained at the individual level and then averaged to generate species-level coloration estimates. These species-level estimates were subsequently used to calculate community-weighted means based on the abundance of each taxon in each stream.

Wing coloration was not quantified because most species sampled in the study had predominantly transparent or nearly transparent wings, and our protocol was designed specifically to quantify body coloration. Quantifying wing pigmentation, transparency, or structural coloration would require a different imaging protocol based on standardized transmitted-light conditions. We nevertheless recognize that wing traits may contribute to thermoregulation and that excluding them limits the scope of our conclusions about whole-body coloration (May 1976).

The percentages of melanin and pruinescence were visually estimated from the calibrated photographs using the same standardized thoracic and abdominal regions adopted for the colour measurements (Guillermo-Ferreira et al. 2023). Melanin was defined as the proportion of each body region covered by dark brown or black pigmentation, whereas pruinescence coverage was defined as the proportion covered by pale, opaque, or bluish-white pruinescence. For the thorax, coverage was estimated across nine standardized areas, each representing approximately one ninth of the evaluated thoracic surface; for the abdomen, coverage was estimated across six areas, each representing approximately one sixth of the evaluated abdominal surface. Coverage was then estimated as the summed proportion of these standardized areas occupied by melanin or pruinescence. The values obtained for the sampled regions were averaged to generate individual-level estimates and subsequently summarized for each species. Because these estimates were obtained visually from external body coloration, they should be interpreted as visible pruinescence coverage rather than direct biochemical quantification of cuticular wax.

After photography, we placed the collected specimens in paper towel envelopes and immersed them in P.A. alcohol (analytical grade) for 24 h (Zygoptera) and 48 h (Anisoptera) (Lencioni 2006). We reexamined all specimens using specialized taxonomic keys and comparisons with reference material (Garrison and von Ellenrieder 2015; Garrison et al. 2006; Lencioni 2005, 2006), and then deposited them in the entomological collection of the Laboratory of Ecology and Conservation (LABECO) at UFPA. We identified specimens to the species level whenever reliable diagnostic characters were available. Individuals lacking the characters necessary for reliable species-level identification were classified as morphospecies to avoid erroneous taxonomic assignments. Morphospecies designations were applied consistently throughout the dataset and represent distinct morphological entities rather than unidentified specimens grouped within a genus.

### Environmental characterization

The same 150-m stream reach used for Odonata sampling was also used for environmental characterization. Abiotic conditions were assessed using the Habitat Integrity Index (HII) assessment protocol (Nessimian et al. 2008). This protocol consists of twelve items that evaluate the pattern of land use adjacent to riparian vegetation; width of the riparian forest and its state of preservation; condition of the riparian forest within a 10 m strip; channel condition regarding sediment type and the presence of retention devices; structure and wear of the stream banks; and characterization of the streambed regarding substrate, aquatic vegetation, debris, and the arrangement of riffle, pool, and meander areas. Each item consists of four to six alternatives ordered to represent increasingly more intact systems. To ensure that each item had the same weight in the analysis, the observed values (ao) were standardized relative to the maximum value for each item (am, Eq. 1). The final index is the mean value for the total characteristics of the sampled habitat (n, Eq. 2). In a recent review, it was one of the most important metrics for explaining the distribution of Odonata in the Amazon (Brasil et al. 2020).

1 $$\:Pi=\:\frac{ao}{am}$$

2 $$\:IIH=\:\frac{{\sum\:}_{i=1}^{n}Pi}{n}$$

Additionally, we measured stream width, canopy cover, mean depth, and air temperature using a thermo-hygrometer, variables frequently associated with the distribution of Odonata (Brasil et al. 2020; Juen et al. 2025). Canopy cover was measured using a densimeter with a convex lens at various sections along the 150-meter sample stretch. The width and depth of the wet area of each stream were measured with a measuring tape at various points along the 150-meter stretch, and the average of the measurements was calculated.

### Data analysis

In our study, each stream was considered an independent sampling unit, totalling 25 sampling units. To assess variation in environmental conditions across sampling sites, we performed a principal component analysis (PCA) using a correlation matrix (Abdi and Williams 2010; Legendre and Legendre 2012). The PCA was performed using the environmental variables HII, canopy cover, stream width, mean depth, and air temperature. To interpret the contribution of environmental variables to each PCA axis, we considered absolute loadings ≥ 0.50 as indicating a strong association with that axis. This threshold was used only for interpretation and did not affect the inclusion of variables in the analysis. Because the environmental variables were measured on different scales, they were z-transformed before analysis (mean = 0, SD = 1). In the PCA ordination, HII was included as one of the environmental variables and therefore contributed to the ordination axes. In contrast, for the PCoA of species composition, HII was not included in the ordination matrix and was used only as a graphical attribute to illustrate the integrity gradient among streams, with higher HII values representing streams with greater environmental integrity. We evaluated variation in Odonata species composition among streams using Principal Coordinates Analysis (PCoA). The analysis was based on the species-abundance matrix, with each stream represented by one independent sampling unit. The abundance data were log-normalized log(x + 1), and pairwise dissimilarities among streams were calculated using the Bray–Curtis index. The PCoA was then performed on the resulting dissimilarity matrix (Legendre and Legendre 2012).

To evaluate how coloration traits varied at the assemblage level, we calculated community-weighted means (CWMs) for each non-circular coloration trait. CWMs summarize the mean trait value of each stream assemblage while accounting for the relative abundance of each species. For each stream, we calculated the community-weighted mean of each coloration trait by averaging species-level trait values weighted by the relative abundance of each species. This procedure generated a site-by-trait matrix in which rows represented streams and columns represented the community-weighted means of Saturation, Value, melanin coverage, and pruinescence coverage. A separate community-weighted mean was calculated for each coloration trait in each stream. For a given stream, the mean species-level trait value was weighted by the relative abundance of each species in that stream, so that more abundant species contributed proportionally more to the resulting community-level value. For each stream (j), the community-weighted mean of trait (k) was calculated as (CWM_{jk}=\sum_{i = 1}^{S_j}p_{ij}t_{ik}), where (p_{ij}) is the relative abundance of species (i) in stream (j), (t_{ik}) is the mean value of trait (k) for species (i), and (S_j) is the number of species recorded in stream (j).

To reduce redundancy between body regions, we averaged the thoracic and abdominal measurements for each coloration trait, generating one whole-body value per individual. We then calculated community-weighted means for Saturation, Value, melanin coverage, and pruinescence coverage in each stream. Using this matrix, we performed a Pearson correlation analysis to avoid including highly correlated coloration traits in the same model (see supplementary material).

We used generalized linear models (GLMs) with Gaussian error distribution to evaluate whether local environmental variables were associated with community-weighted means of coloration traits. In these models, each CWM coloration trait was used as a response variable, and HII, canopy cover, air temperature, and stream width were used as predictor variables. The GLMs were used only to test trait–environment relationships. Variation in species composition among streams was evaluated separately using the PCoA based on the species-abundance matrix.

All analyses were performed in *R* version 4.2.1 (R Core Team 2022), using *RStudio* as the integrated development environment, using the following packages: *ggplot2* (Wickham 2016); *ggrepel* version 0.9.6 (Slowikowski 2024); *ggfortify* (Tang et al. 2016); *ggthemes* (Arnold 2024); *vegan* version 2.6–10 (Oksanen et al. 2017).

## Results

We collected 213 individuals belonging to 32 species or morphospecies. The two most abundant taxa were *Hetaerina laesa* (*n* = 28) and *Mnesarete williamsoni* (*n* = 20) and the full list of taxa and sample sizes is provided in Table S1.

**Fig. 3:**
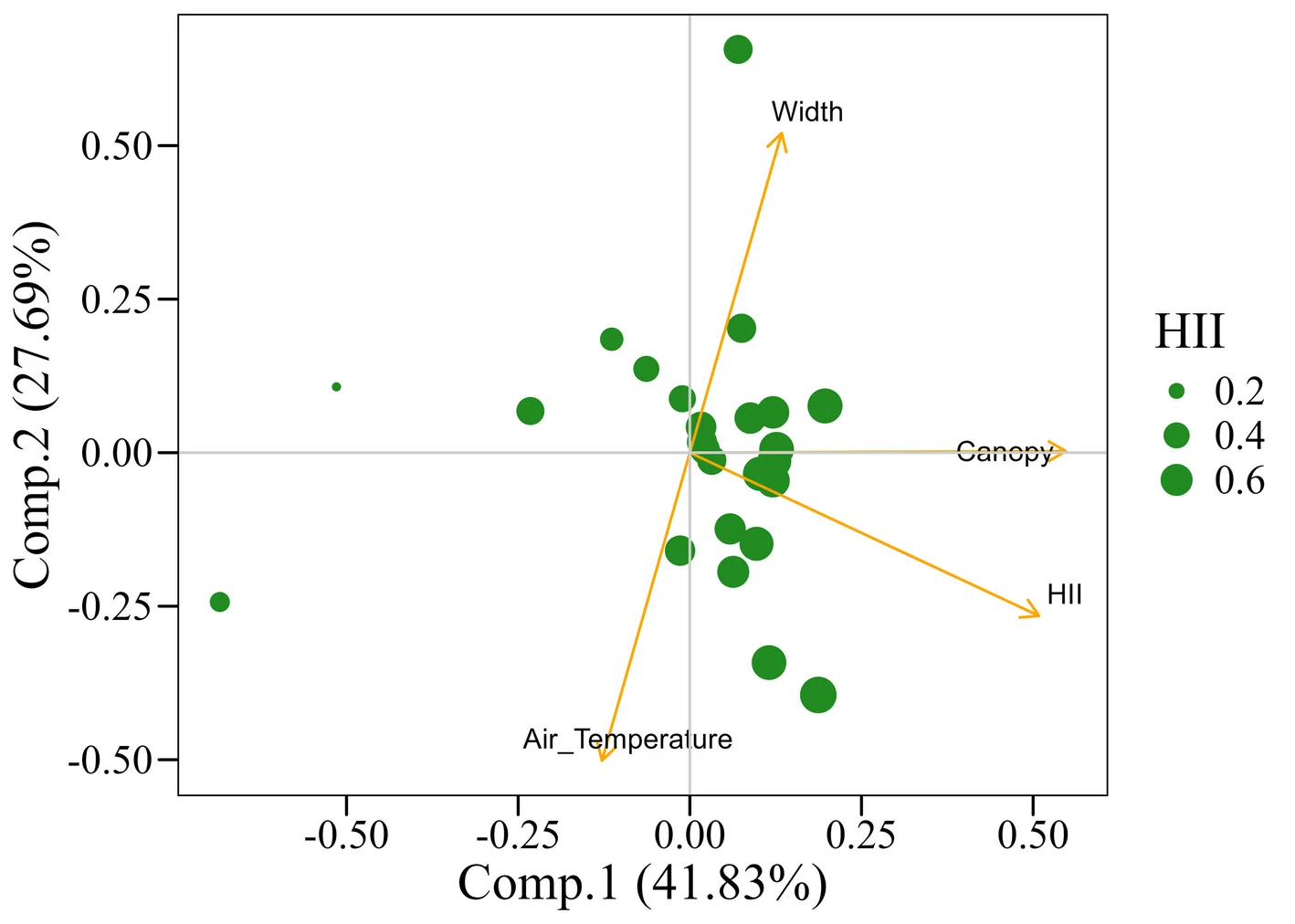
Principal Component Analysis (PCA) of the environmental variables (HII, canopy cover, stream width, mean depth, and air temperature) of the 25 streams sampled in the municipalities of Barcarena and Abaetetuba, Pará, Brazil. Point size is proportional to the Habitat Integrity Index (HII), with larger points representing streams with higher environmental integrity

The first two axes of PCA explained 69.52% of the total variation in the environmental data, with 41.83% explained by the first axis and 27.69% by the second axis (Table 1). The Habitat Integrity Index (HII; loading = 0.854) and canopy cover (loading = 0.920) contributed most strongly to the first axis of the PCA. Stream width showed the strongest association with the second axis (loading = 0.711), whereas air temperature showed only a weak association with this axis (loading = − 0.214; Table 1; Fig. 3). These results indicate that the main environmental gradient was associated primarily with habitat integrity and canopy cover.

**Table 1:** Contribution values of the axes of PCA for the environmental variables air temperature, width, canopy cover, and HII

Variables	Axles PCA	
	Axle1	Axle2
Width	0.173	0.675
Canopy cover	0.711	0.003
Air temperature	-0.166	-0.651
HII	0.660	-0.345
% explanation	41.83	27.69

**Fig. 4:**
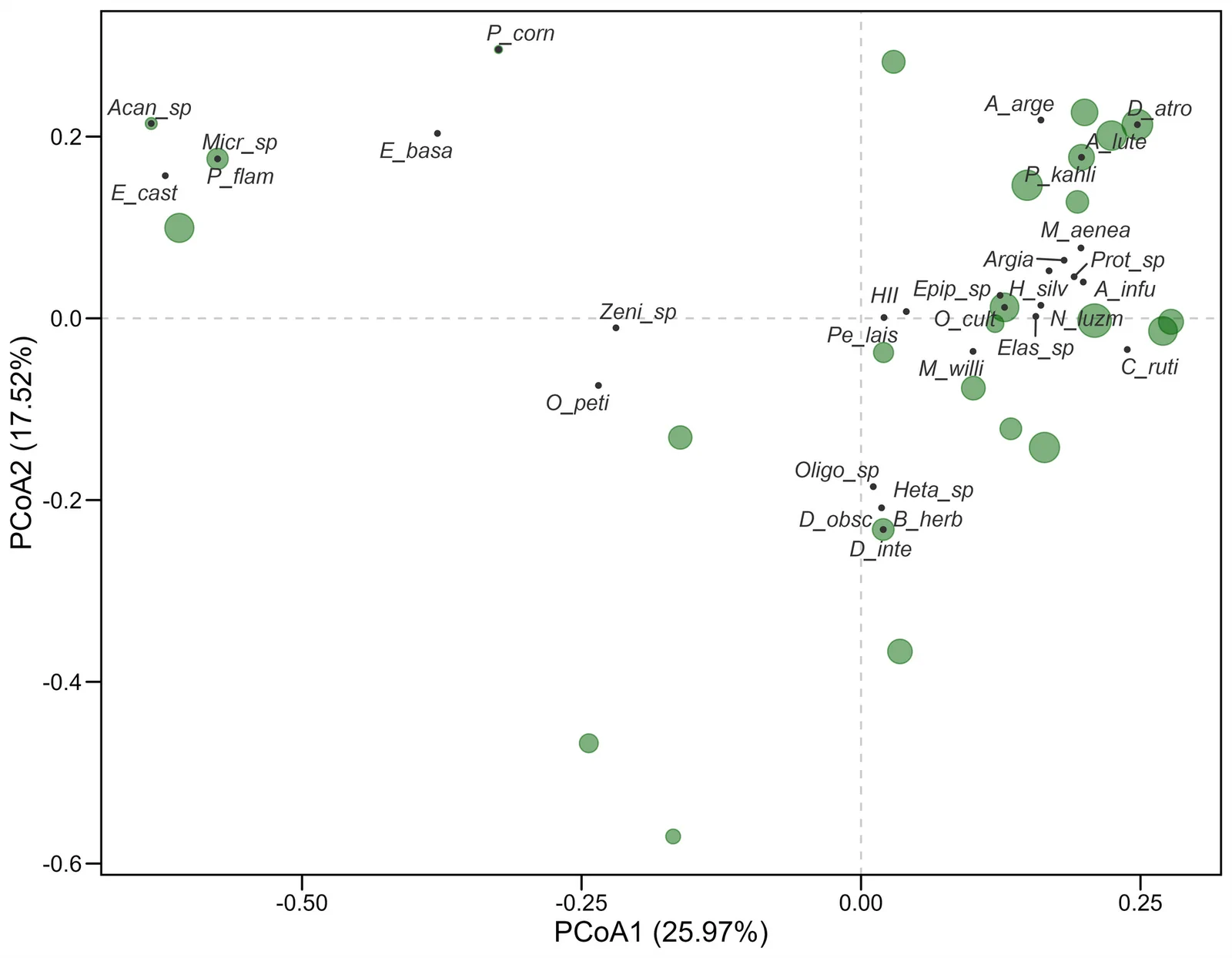
Principal Coordinates Analysis (PCoA) of Odonata species composition (Bray–Curtis dissimilarities of log-normalized abundances) among the 25 streams sampled in the municipalities of Barcarena and Abaetetuba, Pará, Brazil. HII was not included in the ordination; point size is proportional to HII, with larger points representing streams with higher environmental integrity

The PCoA of species composition explained 38.89% of the variation across the first two axes (22.97% on axis 1 and 15.92% on axis 2), indicating compositional variation among streams along the sampled environmental gradient (Fig. 4). *Acanthallagma luteum*, *Chalcopteryx rutilans*, *Dicterias atrosanguinea*, and *Perilestes kahli* occurred only in streams with higher HII values (HII ≥ 0.60), whereas *Acanthagrion kennedii*, *Erythrodiplax basalis*, *Micrathyria* sp., and *Perithemis cornelia* occurred only in streams with lower HII values (HII ≤ 0.53). *Argia infumata* occurred at both ends of the integrity gradient (HII = 0.40–0.79). This pattern indicates compositional turnover between streams with higher and lower environmental integrity.

**Fig. 5:**
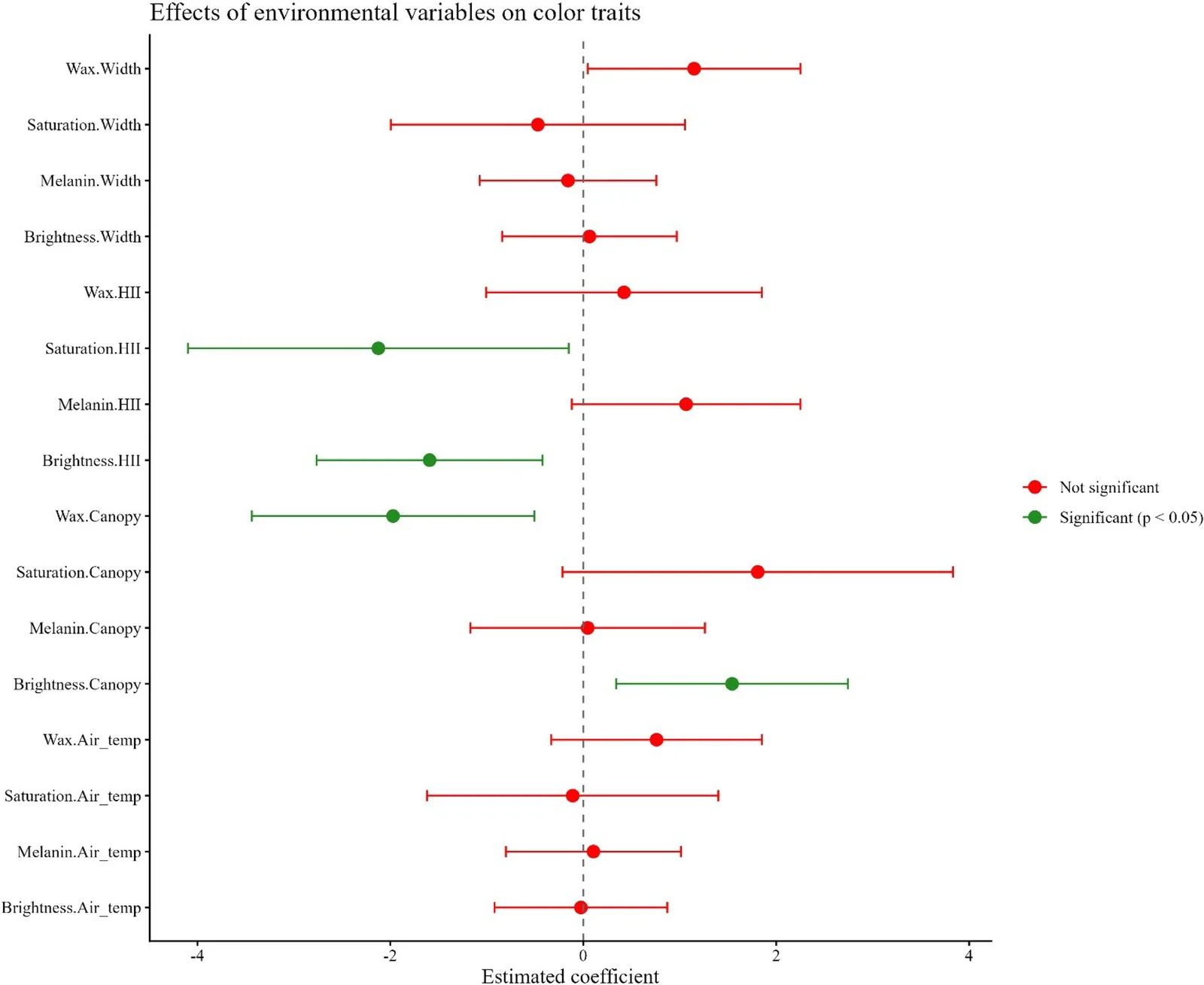
Estimated coefficients from generalized linear models (GLMs) relating community-weighted means of coloration traits (Saturation, Value, melanin coverage, and pruinescence coverage) to standardized environmental variables (air temperature, canopy cover, HII, and stream width). Error bars represent 95% confidence intervals (estimate ± 1.96 SE). Green points indicate statistically significant associations (*p* < 0.05), and red points indicate non-significant associations

Generalized linear models (GLMs) revealed distinct responses of colour traits to environmental variables (Fig. 5). Hue was not included in the GLMs because it is a circular variable and was retained only for descriptive and visual interpretation. Saturation decreased with increasing HII (β = −2.12 ± 1.01 SE; *p* = 0.048), whereas its association with canopy cover was not statistically supported (β = 1.81 ± 1.03 SE; *p* = 0.095). Value increased with canopy cover (β = 1.54 ± 0.61 SE; *p* = 0.020) and decreased with increasing HII (β = −1.59 ± 0.60 SE; *p* = 0.015). pruinescence coverage decreased with increasing canopy cover (β = −1.97 ± 0.75 SE; *p* = 0.016), whereas its association with stream width was not statistically supported (β = 1.15 ± 0.56 SE; *p* = 0.055). Melanin coverage was not significantly associated with HII (β = 1.07 ± 0.60 SE; *p* = 0.093) or with the other environmental variables evaluated (see Table S2 in the supplementary material for more detailed values).

## Discussion

**Fig. 6:**
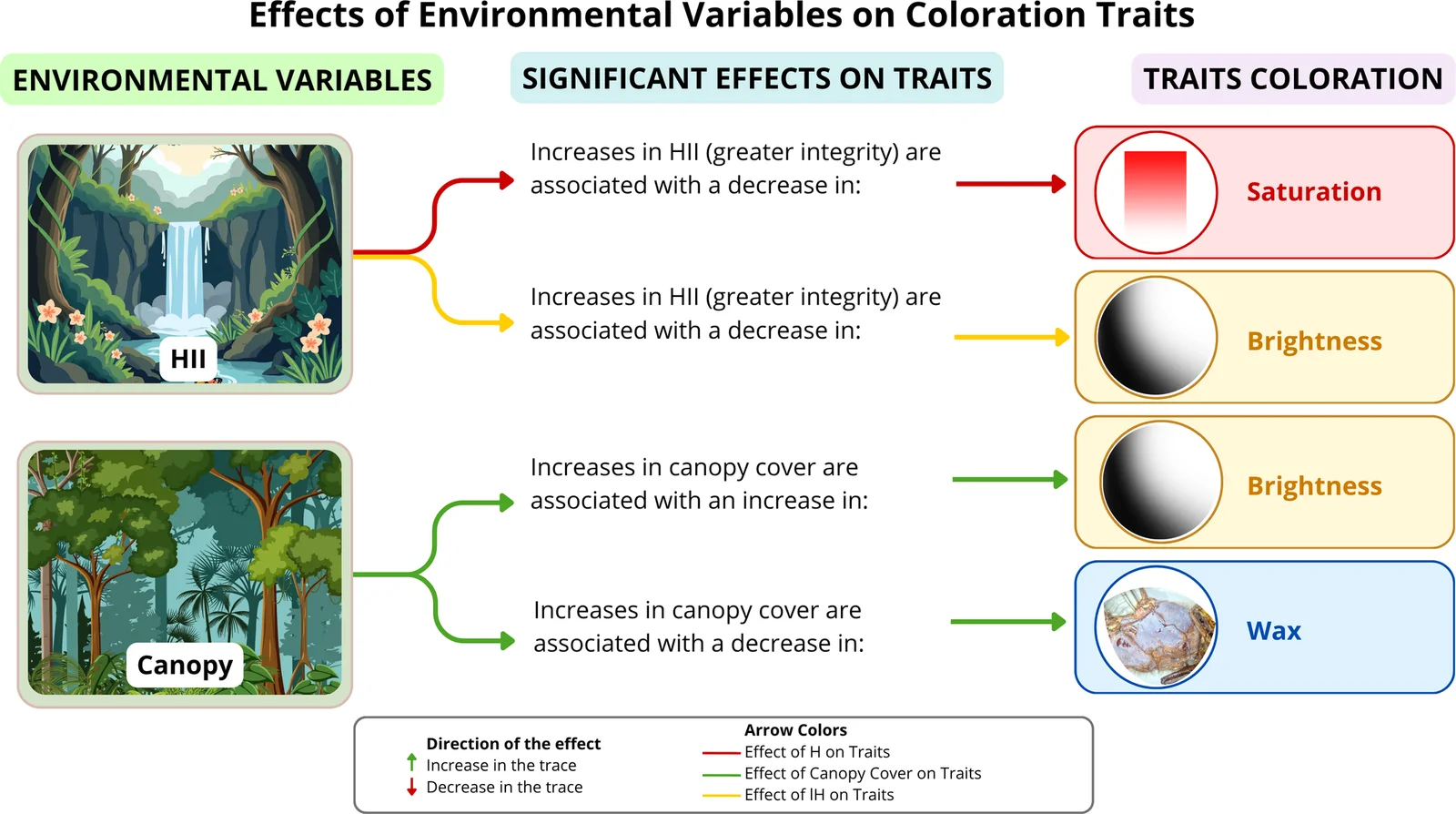
Conceptual summary of the main relationships between environmental gradients (HII and canopy cover) and coloration traits in Odonata assemblages of Amazonian streams. The order of HII and canopy cover is standardized across all columns

Our results indicate that variation in Odonata coloration was associated with local environmental conditions, particularly habitat integrity and canopy cover. These findings are consistent with previous studies showing that habitat structure and light availability can filter Odonata assemblages according to their ecophysiological requirements (May 1976; Oliveira-Junior et al. 2015; Pinkert et al. 2017). Value was associated with both canopy cover and HII, whereas saturation and pruinescence coverage showed more specific responses to the environmental gradient. Similar associations between insect coloration, thermal conditions, and solar exposure have been reported across different climatic and ecological contexts (Clusella-Trullas and Nielsen 2020; Suárez-Tovar et al. 2022). Together, these patterns suggest that coloration may represent one of several functional dimensions through which Odonata assemblages respond to environmental variation, although the observational nature of our study does not allow us to establish the physiological mechanisms underlying these relationships (Fig. 6). Because differences between Anisoptera and Zygoptera were not explicitly tested, the patterns observed for the two suborders should be interpreted descriptively. Our results primarily reflect community-level responses of coloration traits to environmental gradients and do not provide a formal test of differences between suborders.

Coloration, associated with thermoregulation, communication, and territory defense, may represent a functional component sensitive to environmental changes. In more shaded streams, assemblages showed higher community-weighted Value, which contrasts with our prediction that reduced solar exposure would favour darker assemblages. In more open environments, assemblages showed a higher percentage of pruinescence, a characteristic associated with light reflection, UV-related optical properties, and visual signalling. The pruinescence pattern is consistent with studies indicating that reflective structures may contribute to optical responses in areas with higher solar incidence, whereas the Value pattern does not match the expectation that darker tones favour heat gain in cooler environments (Suárez-Tovar et al. 2022; Pinkert et al. 2017; Stelbrink et al. 2019; Heidrich et al. 2018). Darker body surfaces may absorb more solar radiation and thereby facilitate heat gain in Odonata (May 1976).

Hue was interpreted only descriptively because it is a circular component of colour, in which values close to 0° and 360° represent similar chromatic categories. For this reason, Hue was not included in the GLM analyses and was not used to support inferential conclusions about trait–environment relationships. Instead, Hue was used only to describe general chromatic variation among species, while ecological interpretations were based on Saturation, Value, melanin coverage, and pruinescence coverage.

Saturation and Value describe different dimensions of coloration. Saturation indicates chromatic purity, whereas Value represents the relative lightness of the body surface (Evans and Swenholt 1967). Their distinct responses to HII and canopy cover suggest that environmental filtering does not act on body coloration as a single trait. Instead, different components of coloration may respond independently to local light regimes, background contrast, and thermal conditions (Badiane et al. 2022).

Melanin is generally associated with greater absorption of solar radiation and may facilitate warming under cool or shaded conditions (Slominski et al. 2004). Nevertheless, melanin was not significantly associated with the environmental variables evaluated in this study. This lack of a clear relationship may reflect melanin’s multiple functions, including thermal regulation, mechanical protection, immune defense, and visual signaling, as well as the contributions of behavioral thermoregulation and phylogenetic differences among species (Okude and Futahashi 2021).

The positive association between Value and canopy cover suggests that brighter coloration may be more prevalent in shaded streams (Dalrymple et al. 2018). This pattern does not support a simple thermal-melanism interpretation and suggests that melanin-based coloration may not be acting primarily as a heat-absorption trait in this system. Instead, darker pigmentation may also be related to photoprotective functions or other physiological roles, depending on local radiation exposure and species-specific traits. Conversely, the negative association between Value and HII indicates that lower-integrity streams supported assemblages with higher community-weighted Value. These patterns may reflect differences in the thermal and light environments among streams, although the mechanisms underlying these relationships require further investigation (Badiane et al. 2022).

pruinescence coverage also emerged as a relevant coloration trait associated with environmental variation. Because this trait was estimated visually from calibrated photographs, it should be interpreted as visible pruinescence rather than direct biochemical quantification of cuticular wax. Species-level values of the measured coloration traits are provided in the supplementary material (Table S5), but differences between Anisoptera and Zygoptera were not formally tested. Therefore, we avoid interpreting pruinescence as a suborder-level pattern and focus instead on community-level associations with environmental gradients.

pruinescence should not be interpreted as a mechanism that increases heat absorption. Its reflective properties may instead be associated with UV reflection and optical signalling, rather than with thermal regulation or protection against water loss. Thus, greater pruinescence coverage in species associated with more exposed environments may reflect differences in UV-reflectance and visual signalling under higher solar exposure, rather than promote body warming (Sheikh et al. 2017). This interpretation is consistent with the greater exposure to solar radiation expected in streams with reduced canopy cover.

These environmental conditions may impose different thermoregulatory pressures on Odonata species. Although we expected melanin to be associated with environments with lower canopy cover and higher temperature, as predicted by the thermal melanism paradigm and by studies linking darker pigmentation to heat absorption and thermoregulation in insects and Odonata (May 1976; Clusella-Trullas et al. 2007; Pinkert and Zeuss 2018), melanin coverage was not significantly associated with the environmental variables evaluated. One possibility is that the thermal amplitude of the sampled environments was insufficient to elicit detectable melanin responses. Alternatively, Value (V) may better reflect variation in the local light environment in these streams, whereas pruinescence may be more closely related to UV reflection, optical properties, and visual signalling than to thermal modulation. However, melanin coverage showed only a weak, non-significant negative correlation with Value (*r* = − 0.301; *n* = 25), and this relationship should be interpreted cautiously because lightness measures can be influenced by multiple pigmentary and structural components. In addition, the dark areas visually classified as melanin may also include other pigments, such as ommochromes, which were not chemically quantified in our study (Suárez-Tovar et al. 2022). These findings reinforce the need for further experimental studies that directly investigate the relationship among color, temperature, and physiology in Amazonian dragonflies.

Melanin-based pigmentation may influence thermoregulation by increasing the absorption of solar radiation and contributing to darker body coloration (Clusella-Trullas et al. 2007; Pinkert and Zeuss 2018). However, melanin coverage and Value represent different components of coloration: the former quantifies the proportion of the body covered by dark pigmentation (Slominski et al. 2004; Wakamatsu and Ito 2023), Value is a digital descriptor of relative lightness within the HSV colour space and should not be interpreted as a direct spectrophotometric measure of light reflectance. The relationship between melanin coverage and Value should therefore be interpreted cautiously, because lightness-related metrics can be influenced by multiple pigmentary and structural components, including melanin, ommochromes, pruinescence, and cuticular properties (Lopez et al. 2024). Because our study did not evaluate pigment chemistry, pathogen pressure, humidity, or immune function, we avoid interpreting melanin coverage in relation to immune defense or Gloger-like mechanisms.

Dragonflies exhibit substantial variation in body coloration, which may reflect multiple ecological, behavioral and physiological functions (Suárez-Tovar et al. 2022). Many Anisoptera species occur in more open and sunlit habitats, where solar radiation is important for thermoregulation (May 1976; Worthen and Guevara-Mora 2024). In our study, *Erythrodiplax basalis* and *Micrathyria* sp., which occurred only in streams with lower integrity, showed relatively high pruinescence coverage (Table S3). Because differences between suborders were not formally tested, we interpret this pattern descriptively.

Zygopterans are associated with more intact environments, with denser riparian vegetation along the margins and more closed canopy and environments with few physical alterations along channel morphology (Albuquerque et al. 2024). At the community level, variation in coloration traits was associated with differences in habitat integrity and canopy cover among streams. However, our analyses do not allow us to determine whether the coloration of individual species directly reflects their specific habitat requirements (Brito et al. 2024).

Overall, our results do not identify a single coloration trait associated with high-quality streams. Instead, the environmental gradient filtered individual components of coloration differently. Greater habitat integrity was associated with lower Saturation and Value, whereas canopy cover was positively associated with Value and negatively associated with pruinescence coverage. These patterns suggest that coloration in Odonata assemblages may reflect multiple, non-exclusive mechanisms. Darker and less saturated coloration in more preserved and shaded streams may be consistent with behavioural use of both sunlit patches and shaded refuges, camouflage in darker forest environments, and visually mediated interactions (Cezário et al. 2021). In addition, Gloger-like mechanisms could theoretically favour darker pigmentation in warmer and more humid environments through links with pathogen pressure and immune defense, but this hypothesis was not evaluated here. Conversely, higher Value may reflect greater light reflection or colours that are more effective for intra-specific communication, including territorial and sexual signalling, under particular light environments. Greater pruinescence coverage in more exposed streams should be interpreted primarily as a UV-related optical trait, potentially contributing to UV reflection and visual signaling, rather than as a thermal mechanism. These findings indicate that the ecological meaning of coloration depends on the trait considered and that Saturation, Value, melanin coverage, and pruinescence coverage should not be treated as interchangeable indicators of a single functional mechanism. Rather, these traits may be associated with different combinations of optical, photoprotective, behavioural, and, in some cases, thermoregulatory functions.

## Conclusion

The results of our study show that environmental gradients are associated with variation in multiple coloration traits in Odonata assemblages. Habitat integrity and canopy cover were related to variation in Saturation, Value, and pruinescence coverage, indicating that these traits may respond differently to local environmental conditions. In contrast, melanin coverage showed no clear association with the environmental variables evaluated, suggesting that other physiological, behavioural, or structural mechanisms may also influence coloration patterns in the studied assemblages. Because we treated Hue only descriptively and averaged thoracic and abdominal values, our conclusions refer to non-circular whole-body coloration traits at the community level.

The observed associations between coloration traits and habitat integrity also suggest a potential application for conservation assessment. If consistent relationships between coloration and environmental conditions are confirmed across regions and species, coloration traits could contribute to identifying dragonfly species that may be more vulnerable to habitat degradation or changes in canopy cover. Trait-based information could therefore complement occurrence and abundance data when prioritizing species or assemblages for monitoring. However, this potential application requires validation using independent datasets and species-level analyses that directly test whether coloration traits predict sensitivity to habitat change. Finally, our results reinforce the need to conserve and restore riparian vegetation in Amazonian streams, as maintaining canopy cover and habitat integrity may help to preserve Odonata assemblages and the diversity of coloration traits associated with them. We recommend that future studies expand investigations of physiological and experimental responses of coloration, as well as their interactions with microclimatic factors, to deepen the understanding of the mechanisms that shape species survival and distribution in tropical aquatic environments.

## Electronic Supplementary Material

Below is the link to the electronic supplementary material.

Supplementary file 1

## Data Availability

Data will be made available on request.
