# Supplementary material for: Habitat integrity and canopy cover shape small-scale variation in coloration among odonate assemblages in Neotropical streams
Source: Oecologia. 2026 Oct 25;208(11):157. doi: 10.1007/s00442-026-06003-3 (PMC13696594; doi:10.1007/s00442-026-06003-3)
Supplement: Supplementary file 1 [file 442_2026_6003_MOESM1_ESM.docx]

**Do environmental gradients filter multiple coloration traits in Odonata assemblages of Neotropical streams?**

OECOLOGIA

Francisco Maciel Barbosa-Santos^1,2*^ (0000-0002-3457-1341), Felipe Datto-Liberato^3^, Rhainer Guillermo-Ferreira^3^, Luciano Fogaça de Assis Montag^1,2^, Thaisa Sala Michelan^1,2^, Leandro Juen^1,2^

^1^Laboratório de Ecologia e Conservação (LABECO), Instituto de Ciências Biológicas (ICB), Universidade Federal do Pará (UFPA), Rua Augusto Corrêa, nº 1, Bairro Guamá, Belém, Pará, CEP 66.075-110, Brazil

^2^Programa de Pós-graduação em Ecologia (PPGECO), Instituto de Ciências Biológicas, Universidade Federal do Pará, Belém, Pará, Brazil

^3^Lestes Lab, Entomology and Experimental Biology Center, Federal University of Triangulo Mineiro, Uberaba, Brazil

*Corresponding author

## [f.macielbarbosa@outlook.com](mailto:f.macielbarbosa@outlook.com) (F. M. Barbosa-Santos)

**Table S1**. List of Odonata species recorded in this study, organized by suborder, with the respective total abundance of photographed individuals.

| **Zygoptera** | **Species** | **Abundance** |
| --- | --- | --- |
|  | *Acanthagrion kennedii* | 3 |
|  | *Acanthallagma luteum* | 2 |
|  | *Argia collata* | 18 |
|  | *Argia gemella* | 4 |
|  | *Argia infumata* | 6 |
|  | *Argia oculata* | 15 |
|  | *Chalcopteryx rutilans* | 7 |
|  | *Dicterias atrosanguinea* | 3 |
|  | *Epipleoneura kaxuriana* | 7 |
|  | *Epipleoneura machadoi* | 17 |
|  | *Hetaerina laesa* | 28 |
|  | *Heteragrion silvarum* | 5 |
|  | *Mnesarete aenea* | 19 |
|  | *Mnesarete williamsoni* | 20 |
|  | *Neoneura luzmarina* | 6 |
|  | *Oxystigma petiolatum* | 3 |
|  | *Perilestes kahli* | 1 |
|  | *Phoenicagrion flammeum* | 1 |
|  | *Protoneura tenuis* | 2 |
| **Anisoptera** |  |  |
|  | *Argyrothemis argentea* | 7 |
|  | *Brachymesia herbida* | 1 |
|  | *Diastatops intensa* | 3 |
|  | *Diastatops obscura* | 1 |
|  | *Elasmothemis sp.* | 2 |
|  | *Erythrodiplax basalis* | 7 |
|  | *Erythrodiplax castanea* | 2 |
|  | *Micrathyria sp.* | 1 |
|  | *Oligoclada pachystigma* | 2 |
|  | *Orthemis cultriformis* | 1 |
|  | *Perithemis cornelia* | 1 |
|  | *Perithemis lais* | 7 |
|  | *Zenithoptera lanei* | 11 |
| **Total** |  | **213** |

**Table S2.** Statistical values for the generalized linear models (GLMs) that assess the effects of stream width, canopy cover, air temperature, and HII on the color traits of dragonflies (saturation, brightness, melanin, and wax).

| **Traits** | **Variables** | **Estimate** | **Std. Error** | **t** | **p** |
| --- | --- | --- | --- | --- | --- |
| Saturation | Width | -0.470 | 0.778 | -0.604 | 0.553 |
| Saturation | Canopy | 1.809 | 1.033 | 1.751 | 0.095 |
| Saturation | Air_temp | -0.109 | 0.770 | -0.142 | 0.889 |
| **Saturation** | **HII** | **-2.124** | **1.007** | **-2.109** | **0.048** |
| Brightness | Width | 0.065 | 0.462 | 0.140 | 0.890 |
| **Brightness** | **Canopy** | **1.543** | **0.613** | **2.518** | **0.020** |
| Brightness | Air_temp | -0.024 | 0.457 | -0.053 | 0.958 |
| **Brightness** | **HII** | **-1.592** | **0.597** | **-2.665** | **0.015** |
| Melanin | Width | -0.158 | 0.468 | -0.338 | 0.739 |
| Melanin | Canopy | 0.045 | 0.620 | 0.073 | 0.942 |
| Melanin | Air_temp | 0.106 | 0.463 | 0.228 | 0.822 |
| Melanin | HII | 1.066 | 0.605 | 1.762 | 0.093 |
| Wax | Width | 1.149 | 0.563 | 2.041 | 0.055 |
| **Wax** | **Canopy** | **-1.972** | **0.747** | **-2.639** | **0.016** |
| Wax | Air_temp | 0.760 | 0.557 | 1.363 | 0.188 |
| Wax | HII | 0.422 | 0.728 | 0.580 | 0.568 |

**Table S3.** Matrix of Pearson correlation coefficients (r) between the functional traits evaluated.

| **Coeficientes de correlação (*r*)** | | | | | |
| --- | --- | --- | --- | --- | --- |
| **Traits** | Hue | Saturation | Brightness | Melanin | Wax |
| Hue | 1 | -0.50056 | -0.37963 | -0.19389 | -0.45054 |
| Saturation | -0.50056 | 1 | 0.081237 | -0.26711 | -0.30756 |
| Brightness | -0.37963 | 0.081237 | 1 | -0.30076 | -0.07503 |
| Melanin | -0.19389 | -0.26711 | -0.30076 | 1 | 0.102656 |
| Wax | -0.45054 | -0.30756 | -0.07503 | 0.102656 | 1 |

**Table S4.** Significance values (p) associated with the Pearson correlation coefficients between the functional traits evaluated.

| **Coeficientes de correlação (*p*)** | | | | | |
| --- | --- | --- | --- | --- | --- |
| **Traits** | Hue | Saturation | Brightness | Melanin | Wax |
| Hue | NA | 0.01082 | 0.061243 | 0.353067 | 0.023811 |
| Saturation | 0.01082 | NA | 0.699475 | 0.196787 | 0.134759 |
| Brightness | 0.061243 | 0.699475 | NA | 0.14405 | 0.721501 |
| Melanin | 0.353067 | 0.196787 | 0.14405 | NA | 0.625341 |
| Wax | 0.023811 | 0.134759 | 0.721501 | 0.625341 | NA |

**Figure S1.** Photograph of dragonflies alongside the *ColorChecker* tool, which helps to balance illumination.

**Table S5.** Measured and evaluated trait values for Odonata specimens regarding melanin, wax, hue, saturation, and brightness value.

| Species | Melanina | Wax | Hue | Saturation | Brightness-value |
| --- | --- | --- | --- | --- | --- |
|  | **Anisoptera** | | | | |
| *Argyrothemis argentea* | 40 | 44 | 155 | 38 | 56 |
| *Brachymesia herbida* | 29 | 12 | 44 | 90 | 48 |
| *Diastatops intensa* | 47 | 22 | 174 | 90 | 32 |
| *Diastatops obscura* | 52 | 27 | 178 | 81 | 20 |
| *Elasmothemis sp.* | 50 | 25 | 55 | 52 | 46 |
| *Erythrodiplax basalis* | 47 | 20 | 207 | 153 | 24 |
| *Erythrodiplax castanea* | 53 | 21 | 138 | 117 | 25 |
| *Micrathyria sp.* | 40 | 44 | 142 | 39 | 61 |
| *Oligoclada pachystigma* | 3 | 4 | 186 | 48 | 24 |
| *Orthemis cultriformis* | 24 | 10 | 147 | 49 | 56 |
| *Perithemis cornelia* | 42 | 25 | 143 | 38 | 52 |
| *Perithemis lais* | 24 | 13 | 29 | 153 | 51 |
| *Zenithoptera lanei* | 34 | 23 | 115 | 50 | 31 |
|  | **Zygoptera** | | | | |
| *Acanthagrion kennedii* | 69 | 12 | 163 | 89 | 40 |
| *Acanthallagma luteum* | 63 | 11 | 161 | 30 | 46 |
| *Argia collata* | 64 | 9 | 95 | 63 | 28 |
| *Argia gemella* | 35 | 10 | 146 | 63 | 42 |
| *Argia infumata* | 52 | 10 | 50 | 46 | 26 |
| *Argia oculata* | 66 | 11 | 167 | 70 | 40 |
| *Chalcopteryx rutilans* | 42 | 9 | 103 | 62 | 34 |
| *Dicterias atrosanguinea* | 23 | 8 | 108 | 121 | 53 |
| *Epipleoneura kaxuriana* | 46 | 8 | 142 | 42 | 50 |
| *Epipleoneura machadoi* | 43 | 8 | 115 | 35 | 59 |
| *Hetaerina laesa* | 43 | 12 | 98 | 68 | 31 |
| *Heteragrion silvarum* | 28 | 7 | 38 | 67 | 56 |
| *Mnesarete aenea* | 52 | 10 | 198 | 66 | 25 |
| *Mnesarete williamsoni* | 56 | 15 | 144 | 124 | 32 |
| *Neoneura luzmarina* | 41 | 9 | 119 | 88 | 50 |
| *Oxystigma petiolatum* | 34 | 19 | 51 | 79 | 41 |
| *Perilestes kahli* | 29 | 8 | 50 | 54 | 74 |
| *Phoenicagrion flammeum* | 20 | 5 | 64 | 29 | 118 |
| *Protoneura tenuis* | 42 | 10 | 142 | 97 | 69 |
